# Role of sexuality in women with chronic pain: Results from an Italian cross-sectional study on chronic headache, fibromyalgia, and vulvodynia

**DOI:** 10.1016/j.ijchp.2024.100472

**Published:** 2024-06-07

**Authors:** Filippo Maria Nimbi, Martina Mesce, Erika Limoncin, Alessia Renzi, Federica Galli

**Affiliations:** Department of Dynamic and Clinical Psychology and Health Studies, Sapienza University of Rome, Via degli Apuli 1, Rome, 00185, Italy

**Keywords:** Nociplastic pain, Fibromyalgia, Headache, Vulvodynia, Genital pain

## Abstract

**Background/objectives:**

To compare sexual functioning, genital pain, and satisfaction among women diagnosed with various Chronic pain (CP) conditions. Additionally, it seeks to explore the role of sexual factors in predicting levels of central sensitization (indicative of CP-related mental and physical distress), physical, and mental quality of life (QoL) for each condition individually.

**Methods:**

From April 2023 to January 2024, 1006 women categorized into five groups (Chronic Headache - CH; Fibromyalgia - FM, Vulvodynia - VU, Comorbidity group - CO, and Healthy Controls - HC) completed an online protocol.

**Results:**

All groups reported sexual impairment: VU group exhibited the highest genital pain prevalence (97.93 %), followed by CO (74.29 %) and FM (55.91 %). ANCOVAs indicated lower sexual functioning scores for FM, VU, and CO compared to HC and CH. VU and CO reported lower satisfaction scores than other groups. Genital pain emerged as the primary predictor of central sensitization across all groups except controls. Regarding mental QoL, sexual satisfaction was significant for CH and CO, while genital pain and sexual satisfaction were significant for VU.

**Conclusion:**

This study emphasizes the importance of integrating genito-pelvic pain assessment and addressing related sexual difficulties in CP diagnostics and care to enhance overall well-being and QoL.

## Introduction

Chronic pain (CP) stands as the foremost prevalent condition globally prompting medical attention, linked to considerable disability accompanied by a substantial psychological and social impact (Cohen et al., 2021). CP, as defined by the International Association for the Study of Pain (IASP), manifests as an unpleasant sensory and emotional ordeal linked with, or resembling, actual or potential tissue damage (Raja et al., 2020). The prevalence rates of CP range from 11 % to 40 %, with a study conducted by the US Centres for Disease Control and Prevention (CDC) estimating the prevalence at 20.4 % (Dahlhamer et al., 2018).

In the classification of the types of CP, special attention has been given in recent years to nociplastic pain (NP) (Fitzcharles et al., 2021; Galli, 2023; Kosek et al., 2021; Nijs et al., 2021). NP describes pain that nociceptive and neuropathic mechanisms cannot explain. It persists for more than 3 months and results from altered function of sensory pathways related to pain in the central and peripheral nervous system, causing in a hypersensitivity to painful stimuli. NP is characteristic of multiple clinical conditions that share common neurophysiologic mechanisms and opens a new framework for understanding the co-occurrence of different chronic disorders and the role of psychological factors related (Fitzcharles et al., 2021).

Under the NP umbrella, research in recent years has produced numerous contributions on Chronic Headache (CH), Fibromyalgia (FM), and Vulvodynia (VU), conditions that are most prevalent in women than men (Caponnetto et al., 2021; Casale et al., 2021; Chisari et al., 2021; Dagostin Ferraz et al., 2024; Lamvu et al., 2021; Martinez-Lavin, 2020; Ruschak et al., 2023).

Headache ranks as the second most significant cause of nonfatal health loss (James et al., 2018). CH is diagnosed when headache attacks persist for more than 15 days per month for a minimum of three months. Recent research suggests a global prevalence of active headache disorders at 52 % (Stovner et al., 2022). Among individuals in the workforce, headaches pose a substantial disability risk, resulting in a considerable economic burden due to both direct and indirect costs.

FM presents as a CP syndrome characterized by widespread musculoskeletal pain typically accompanied by sleep disturbances, fatigue, somatic and cognitive symptoms, as well as mental health conditions (Sarzi-Puttini et al., 2020; Wolfe et al., 2016, 2020). Within the general population, the diagnosis of FM shows a prevalence ranging from 0.2 % to 6.6 %, with significantly higher rates observed in women (ranging from 2.4 % to 6.8 %) and a rising trend in prevalence worldwide (Marques et al., 2017).

VU is a CP syndrome characterized by persistent vulvar pain, described as sharp and burning, occurring without identifiable visible factors or specific clinically identifiable neurological disorders (Bornstein et al., 2016, 2022). Estimates from population-based studies indicate a lifetime prevalence of VU ranging from 8 to 10 % (Harlow et al., 2014). In 2015, new terminology reshaped the classification and definition of persistent vulvar pain, recognizing that multiple variables, including neurological, musculoskeletal, and psychosocial factors, may contribute to it (Bornstein et al., 2016).

What many NP conditions have in common is that they are usually accompanied by other symptoms associated with the central nervous system and strongly related to various clinical psychological factors such as general symptoms (fatigue and cognitive problems), temperamental features (hypersensitivity to environmental stimuli), and psychological symptoms (anxiety, depression) (Fitzcharles et al., 2021). In this sense, it is common to encounter situations of overlap between these clinical conditions (Comorbidity group, CO) in which the person present 2 or more CP manifestations (Fitzcharles et al., 2021; Johnston et al., 2023).

One aspect that remains little explored in CP is the experience related to sexuality. With respect to female sexual health in CP, some studies (Chisari et al., 2021; Connor et al., 2020; Flegge et al., 2023; Granero-Molina et al., 2023; Nimbi et al., 2020) agree that this area is frequently impacted, displaying a high prevalence of sexual dysfunction, and showing a correlation between the severity of pain, psychological issues, and sexual problems. Numerous women experience notably reduced sexual satisfaction linked to elevated levels of pain, pain-related disruptions in daily life, depression, and anxiety (Flegge et al., 2023)

All sexual functioning domains seems to be affected, such as pain during intercourses (Ricoy-Cano et al., 2022), sexual desire (Santos-Iglesias et al., 2022), arousal and orgasm (Solmaz et al., 2016). In another study (Finn et al., 2018), authors compared sexual wellbeing between men and women affected by several CP conditions. Interestingly, important gender differences emerged in terms of variables significantly bearing on sexual functioning. For example, for women age and relationship satisfaction played a central role, while for men only the variable “age” was statistically significant in predicting sexual functioning.

One critical point is that the available literature is mainly focused on one specific conditions, for example sexuality in women with FM (Granero-Molina et al., 2023; Ricoy-Cano et al., 2022; Santos-Iglesias et al., 2022), or studies that collide different CP conditions into macro-groups, without distinguishing the possible specific experience of patients in different diagnoses (Birke et al., 2019; Burri et al., 2014; Piarulli et al., 2021). There is also lack of evidence about the specific role of sexual factors in determining physical and mental QoL, apart from the CP type and pain characterization. Moreover, little is known about the role that sexual experience may play in these women with respect to psychological and physical distress experienced by the individual with CP and quality of life, and whether there may be condition-specific differences in this relationship (Collado-Mateo et al., 2020).

## Aims

The general aim of the present study is to better understand the sexual experiences of women presenting different conditions under CP (namely, CH, FM, VU and CO) to identify and discuss similarities and differences. Specifically, we aim to:

(i) Investigate the presence and characteristics of genito-pelvic pain and explore possible significant differences in sexual functioning and satisfaction in the four groups compared with healthy controls from the general population.
(ii) Investigate the role of sexual variables (functioning, genito-pelvic pain, and satisfaction) associated to the levels of CP related distress, physical, and mental QoL separately for each group.

Our hypotheses were that each group would show a similar situation of impairment concerning sexual experience, with peculiarities. For example, we expect to find higher levels of genito-pelvic pain and impairment of sexual functioning in VU, FM and CO groups in line with the literature (Casale et al., 2021; Chisari et al., 2021; Collado-Mateo et al., 2020; Nimbi et al., 2020; Ricoy-Cano et al., 2022), significantly different from CH and HC (Gordon et al., 2014; Nappi et al., 2012; Nimbi et al., 2020).

## Materials and methods

### Procedures

The data utilized in this study were gathered as part of a broader research endeavour aimed at evaluating specific psychological factors potentially affecting the experience of CP in women. These factors encompassed experiences of trauma, psychological distress, personality traits, mental pain, sensitivity to environmental stimuli, and impacts on sexual well-being. A total of 1237 Italian women took part in the study, recruited through a snowball sampling technique involving associations for CH, FM, and VU patients, who disseminated the web survey via their official websites and social media platforms such as Facebook, Instagram, X, and LinkedIn. The survey was conducted online using Google Forms, with data collection spanning from April 2023 to January 2024. Prior to participating, individuals were required to provide informed consent and furnish details regarding their CP diagnosis, including the year of diagnosis and the healthcare professional or institution responsible for it. Participant anonymity was ensured, and no compensation was provided for their involvement. Ethical clearance for the project was obtained from the ethical committee of the Department of Dynamic and Clinical Psychology and Health Studies, Sapienza University of Rome on November 25, 2022 [Protocol number 0001979 UOR: SI000092—Classified VII/15]. Eligible participants identified as cisgender women, were at least 18 years old, proficient in Italian, and had received a diagnosis of CM, FM, and/or VU from a specialist physician (neurologist or rheumatologist) at least six months prior. After excluding ninety-three responses (7.52 %) that did not meet the inclusion criteria or were duplicate entries, the final cohort comprised 1144 participants who completed the mandatory survey. Assessments pertaining to sexuality were included in the optional section of the protocol, which was completed by 1006 out of the total 1144 participants. For a comprehensive description of the self-report protocol, please refer to the "measures" section. Sociodemographic characteristics of the participants analysed in this study pertain to the 1006 women who completed the voluntary section and are summarized in Table 1.

**Table 1:** Sociodemographic data and description of the participants (*n* = 1006).

	Total Group (*n* = 1006)	Chronic Headache (CH) (*n* = 195)	Fibromyalgia (FM) (*n* = 186)	Vulvodynia (VU) (*n* = 193)	Comorbidity (CO) (*n* = 315)	Healthy Controls (HC) (*n* = 117)	Significance
	*M* ± SD	*M* ± SD	*M* ± SD	*M* ± SD	*M* ± SD	*M* ± SD	F (4, 1001)
Age	40.71±13.31	39.92 ± 12.39	46.58 ± 12.15	32.36 ± 10.99	44.40 ± 12.52	36.50±13.8	43.519 df = 4 *p* < 0.001
	**n (%)**	**n (%)**	**n (%)**	**n (%)**	**n (%)**	**n (%)**	**Chi-squared**
Sexual Orientation							26.908 df = 12 *p* = 0.008
Heterosexual	923 (91.75)	174 (89.23)	179 (96.24)	171 (88.60)	295 (93.65)	104 (88.89)	
Bisexual, Pansexual or Polisexual	59 (5.86)	13 (6.67)	3 (1.61)	13 (6.74)	18 (5.71)	12 (10.26)	
Lesbian	14 (1.39)	4 (2.05)	4 (2.15)	5 (2.59)	0	1 (0.85)	
Asexual spectrum	10 (0.99)	4 (2.05)	0	4 (2.07)	2 (0.63)	0	
**Civil status**							102.258 df = 16 *p***<** 0.001
Single	382 (37.97)	77 (39.49)	44 (23.66)	104 (53.89)	95 (30.16)	62 (52.99)	
Married/Civil Union	378 (37.57)	78 (40.00)	96 (51.61)	34 (17.62)	142 (45.08)	28 (23.93)	
Separated/Divorced	81 (8.05)	12 (6.15)	18 (9.68)	7 (3.63)	36 (11.43)	8 (6.84)	
Widowed	8 (0.80)	0	2 (1.08)	1 (0.52)	4 (1.27)	1 (0.85)	
Cohabitant	157 (15.61)	28 (14.36)	26 (13.98)	47 (24.35)	38 (12.06)	18 (15.38)	
**Current relational status**							- *p***=** 0.134
Single	210 (20.87)	32 (16.41)	40 (21.51)	34 (17.62)	73 (23.17)	31 (26.50)	
Monogamous couple	782 (77.73)	157 (80.51)	143 (76.88)	158 (81.87)	239 (75.87)	85 (72.65)	
Polyamorous/ non-monogamous relationship	14 (1.39)	6 (3.08)	3 (1.61)	1 (0.52)	3 (0.95)	1 (0.85)	
**Education degree**							72.374 df = 16 *p***<** 0.001
Primary school	1 (0.10)	0	0	1 (0.52)	0	0	
Middle school	72 (7.16)	8 (4.10)	23 (12.37)	3 (1.55)	32 (10.16)	6 (5.13)	
High school	420 (14.75)	78 (40.00)	90 (48.39)	69 (35.75)	153 (48.57)	30 (25.64)	
Degree	387 (38.47)	83 (42.56)	61 (32.80)	92 (47.67)	97 (30.79)	54 (46.15)	
Post-degree	126 (12.52)	26 (13.33)	12 (6.45)	28 (14.51)	33 (10.48)	27 (23.08)	
Work Status							88.604 df = 12 *p*=< 0.001
Unemployed	193 (19.18)	47 (24.10)	40 (21.51)	20 (10.36)	76 (24.13)	10 (8.55)	
Student	136 (13.52)	25 (12.82)	7 (3.76)	50 (25.91)	4 (7.62)	30 (25.64)	
Employed	629 (62.52)	117 (60.00)	124 (66.67)	120 (62.18)	197 (6.54)	71 (60.68)	
Retired	48 (4.77)	6 (3.08)	15 (8.06)	3 (1.55)	18 (5.71)	6 (5.13)	
**Socio-economic status**							- *p* = 0.070
Low	112 (11.13)	18 (9.23)	26 (13.98)	17 (8.81)	43 (13.65)	8 (6.84)	
Middle-Low	269 (26.74)	45 (23.08)	55 (29.57)	47 (24.35)	95 (30.16)	27 (23.08)	
Middle	542 (53.88)	108 (55.38)	93 (50.00)	112 (58.03)	160 (50.79)	69 (58.97)	
Middle-High	78 (7.75)	22 (11.28)	12 (6.45)	15 (7.77)	16 (5.08)	13 (11.11)	
High	5 (0.50)	2 (1.03)	0	2 (1.04)	1 (0.32)	0	

Note: CO group specifically includes 183 participants with comorbid diagnoses of FM and CH; 44 of FM and VU; 33 of CH and VU; 55 of FM, CH, and VU.

### Participants

The average age of the study participants was 40.71 ± 13.31 years, ranging from 18 to 75 years. They primarily identified as heterosexual and were involved in monogamous relationships. The majority possessed moderate to moderately high levels of education, equivalent to a high school diploma or University Degree, with nearly 63 % currently employed. White Caucasians constituted the predominant ethnic background (98.51 %), and they mainly resided in small towns or cities with a medium to medium-low socioeconomic status. All participants had received a CP diagnosis between 1982 and 2022, predominantly from specialized physicians such as neurologists, rheumatologists, and gynaecologists. Participants were categorized into five groups based on their declared diagnoses: chronic headache (CH), fibromyalgia (FM), vulvodynia (VU), comorbidity group (CO; when participants had two or more diagnoses among CH, FM, and VU), and healthy controls (HC; women who reported no history of CP related to the three diagnoses considered for the study).

### Measures

The protocol encompassed self-report measures designed to explore specific psychological variables in accordance with the objectives of the research project. To streamline administration and prevent undue participant burden, the protocol was divided into a mandatory section, completed by 1144 participants, and an optional section, completed by a total of 1006 women. The time required for assessment was approximately 30 min for the mandatory portion and an additional 10 min for the voluntary one.

The questionnaires utilized in this study include:

Sociodemographic Questionnaire – Participants were asked to complete a brief sociodemographic survey aimed at collecting general information such as age, gender, sexual orientation, marital status (the legally defined marital state based on laws or customs of the country), relationship status (description of the type of relationship the person is in, if any), educational level, employment status, socioeconomic status, ethnicity, residential location, and relevant details regarding the diagnosis of CP conditions.

Central Sensitization Inventory (CSI) (Chiarotto et al., 2018; Mayer et al., 2012) – This assessment was developed to evaluate the overlapping symptomatic aspects of central sensitivity syndrome. It serves as a preliminary screening tool to identify the presence of the syndrome and to alert clinicians to potential symptom-related connections. The CSI is hypothesized to reflect psychological hypervigilance and burden than increased responsiveness of nociceptive neurons more closely (Adams et al., 2023). In this sense, it was considered to be more reflective of the psychological and physical distress experienced by the individual with CP. The inventory consists of two sections: part A generates a total score ranging from 0 to 100 for 25 items relating to current health symptoms, with response options on a scale from never = 0 to always = 4; part B investigates whether patients have previously been diagnosed by a physician with any of seven distinct conditions. The CSI has demonstrated satisfactory validity among CP patients, with higher scores indicating a greater manifestation of central sensitivity or CP related distress. The Cronbach's alpha for this measure in the current study was 0.923.

Short Form (SF-12) - Quality of Life Assessment (Ware et al., 1996): The SF-12, derived from the original SF-36, is a concise generic health survey designed to assess both physical and psychological quality of life. It provides two summary measures for self-assessment of physical and mental health ranging from 0 (very bad QoL) to 100 (very good QoL), which are interchangeable with the SF-36 outcomes (Ware & Gandek, 1998). Improved scores reflect a higher level of quality of life in the respective domain. The Cronbach's alpha for this measure in the current study was 0.846 and 0.723 respectively, for physical and psychological QoL.

The Female Sexual Functioning Index (FSFI) (Filocamo et al., 2014; Rosen et al., 2000) – This widely-used measure assesses sexual functioning in sexually active women over the past four weeks, utilizing a 19-item Likert scale ranging from 0 to 5 across six sexual domains: desire, arousal, lubrication, orgasm, satisfaction, and pain. Psychometric studies have reported good reliability, validity, and the ability to discriminate between women with or without sexual dysfunctions. A total score below 26.55 indicates the presence of sexual dysfunction. The Cronbach's alpha for this measure in the current study was 0.932 for the total score, 0.920 for desire, 0.919 for arousal, 0.733 for lubrication, 0.907 for orgasm, 0.864 for satisfaction and 0.929 for pain.

The Short Form McGill Pain Questionnaire (SF-MPQ) (Melzack, 1987): Comprising 15 descriptors (11 sensory and 4 affective), this questionnaire assesses pain intensity on a scale from "none" to "severe." It gathers both quantitative and qualitative information about the subjective experience of pain. A total score can be calculated as an indicator of pain intensity. For this study, the adapted version for genito-pelvic pain by Nimbi et al. (2020) was used, focusing on pain localized in the genital area and incorporating the Marinoff scale for dyspareunia (Marinoff & Turner, 1992). The Cronbach's alpha for this measure in the current study was 0.935.

The Sexual Satisfaction Scale (SSS) - (Meston & Trapnell, 2005) (Nimbi et al., under review): This 30-item measure evaluates sexual satisfaction across five factors: contentment, communication, compatibility, relational, and personal concern. It has demonstrated good psychometric properties and discriminative capability between clinical and nonclinical populations. In this study, only the contentment subscale (6 items, ranging from 6 to 30) was employed, applicable to all individuals regardless of partnership status. The Cronbach's alpha for this measure in the current study was 0.849.

### Statistical analysis

First, sociodemographic descriptive data were discussed, highlighting the characteristics of women reporting CP divided into five groups (CH, FM, VU, CO and HC). Due to the difference among groups and the possible impact on sexual functioning and CP manifestation, age was defined as covariates for every analysis of variance (ANOVA) and regression model included in the present study. Secondly, qualitative and quantitative characteristics of genital and sexual pain were analysed comparing the five groups using chi squared analysis for frequences and factorial one-way analysis of covariances (ANCOVAs). To analyse the differences in sexual domains between groups of women reporting CP, ANCOVAs and multivariate analysis of covariances (MANCOVAs) were conducted. To better explore the role of sexuality, Bonferroni Post Hoc analyses were run to specify the differences between groups. Following the last aim of the current study, sexual predictors of central sensitization (indicative of CP-related mental and physical distress) and QoL (physical and mental) were run separately for each group using hierarchical multiple regressions. All data were analysed using Jamovi version 2.4.11.

## Results

To ensure a sufficient statistical power (0.80), a predetermined minimum of 269 participants was calculated a priori for the ANCOVA with GPower 3.1. These analyses involved five groups and one covariate and required a minimum effect size of 0.25. The effective sample size for the ANCOVA analyses ultimately reached 532 participants for FSFI scores (resulting in a post-hoc observed statistical power of 0.99) and 1006 for SSS-CD score (resulting in a post-hoc observed statistical power of 1).

Regarding hierarchical multiple regressions, aiming to a minimum statistical power (0.80), a predetermined number of 43 participants for each group was calculated a priori. These analyses involved three predictors and one covariate and required a minimum effect size of 0.25. The final sample size for these analyses reached a minimum of 68 participants for HC (resulting in a post-hoc observed statistical power of 0.94) and a maximum of 144 for CO group (resulting in a post-hoc observed statistical power of 1).

Table 1 reports descriptive statistics of the sociodemographic variables assessed for the current study among groups. The groups differed significantly in age (*F* = 43.519; df = 4; *p* < 0.01). Bonferroni's post hoc comparisons specified that FM and CO are significantly older than CH and HC, which in turn are older than VU. This evidence justifies the choice to include age as a covariate in subsequent analyses. In addition, the groups differ significantly in sexual orientation, marital status, education, and work status. Again, age difference may have played a significant role in these differences, with older participants being more often married, with higher educational levels, and employed or retired.

Following the first aim, a description of the characteristics of genito-pelvic pain divided by group is given in Table 2. A first significant difference emerges among women “Declaring having had pain in the genital/sexual area in the last 6 months”. The chi-squared test is significant and shows that the group with vulvodynia reports pain in 97.93 % of cases, compared with 74.29 % in the CO group and 55.91 % in FM. The rate of women reporting pain by the CH group is slightly higher than that of HCs (38.97% vs 32.48 %).

**Table 2:** Qualitative and quantitative characteristics of genital and sexual pain.

		Chronic Headache (CH) (*n* = 195)	Fibromyalgia (FM) (*n* = 186)	Vulvodynia (VU) (*n* = 193)	Comorbidity (CO) (*n* = 315)	Healthy Controls (HC) (*n* = 117)	
Variable		n (%)	n (%)	n (%)	n (%)	n (%)	Sign.
**Declaring having had pain in the genital/sexual area in the last 6 months**		76 (38.97)	104 (55.91)	189 (97.93)	234 (74.29)	38 (32.48)	218.848 df=4 *p* < 0.001
		**n (%)**	**n (%)**	**n (%)**	**n (%)**	**n (%)**	
Localisation of the pain*	Vaginal introitus (opening, entrance)	35 (47.95)	50 (49.50)	163 (86.24)	161 (70.61)	21 (56.76)	61.565 df=4 *p* < 0.001
	Initial part of the vagina	35 (47.95)	41 (40.59)	118 (62.43)	127 (55.70)	17 (45.95)	15.012 df=4 *p* = 0.005
	Deep part of the vagina	29 (39.73)	44 (43.56)	76 (40.21)	96 (42.11)	14 (37.84)	- *p* = 0.960
	External genitalia (vulva, labium majora and minora)	17 (23.61)	33 (32.67)	122 (64.55)	118 (51.75)	13 (35.14)	51.095 df=4 *p* < 0.001
	Ovary	23 (31.51)	30 (29.70)	35 (18.52)	76 (33.33)	8 (21.62)	13.042 df=4 *p* = 0.011
	Uterus	18 (24.66)	22 (21.78)	25 (13.23)	62 (27.19)	8 (21.62)	12.479 df=4 *p=* 0.014
	Clitoris	8 (10.96)	18 (17.82)	78 (41.27)	67 (29.39)	8 (21.62)	32.743 df=4 *p* < 0.001
	Anal area (perineum and anus)	10 (13.70)	27 (26.73)	63 (33.33)	74 (32.46)	8 (21.62)	12.583 df=4 *p* = 0.014
	Urethra	6 (8.33)	13 (13.00)	46 (24.34)	50 (22.42)	7 (18.92)	12.372 df=4 *p* = 0.015
		***M*±DS**	***M*±DS**	***M*±DS**	***M*±DS**	***M*±DS**	
**Genital Pain Duration**	Months	34.39±11.54	53.88±9.55	60.61±5.60	71.93±5.77	32.52±17.03	3.054 df=4 *p* = 0.017
		**n (%)**	**n (%)**	**n (%)**	**n (%)**	**n (%)**	
**Pain changes with time**	Brief momentary transient	45 (59.21)	35 (33.65)	50 (26.46)	51 (21.79)	21 (55.26)	56.853 df=8 *p* < 0.001
	Rhythmic periodic intermittent	15 (19.74)	25 (24.04)	73 (38.62)	75 (32.05)	9 (23.68)	
	Continuous steady constant	16 (21.05)	44 (42.31)	66 (34.92)	108 (46.15)	8 (21.05)	
		***M*±DS**	***M*±DS**	***M*±DS**	***M*±DS**	***M*±DS**	
**Marinoff scale**	Mean score	1.46±0.09	1.79±0.08	2.23±0.06	1.99±0.05	1.58±0.13	13.359 df=4 *p* < 0.001
		**n (%)**	**n (%)**	**n (%)**	**n (%)**	**n (%)**	
	Pain with intercourse that doesn't prevent the completion	30 (39.47)	32 (30.77)	21 (11.11)	50 (21.37)	15 (39.47)	67.590 df=12 *p* < 0.001
	Pain with intercourse requiring interruption or discontinuance	30 (39.47)	41 (39.42)	103 (54.50)	112 (47.86)	13 (34.21)	
	Pain with intercourse preventing any intercourse	7 (9.21)	24 (23.08)	63 (33.33)	65 (27.78)	6 (15.79)	
	No pain with intercourse	9 (11.84)	7 (6.73)	2 (1.06)	7 (2.99)	4 (10.53)	
		**n (%)**	**n (%)**	**n (%)**	**n (%)**	**n (%)**	
**FSFI sexually dysfunctional**	No	49 (46.67)	31 (34.07)	25 (20.16)	35 (24.31)	38 (55.88)	38.867 df=4 *p* < 0.001
**(Total score < 26.55)**	Yes	56 (53.33)	60 (65.93)	99 (79.84)	109 (75.69)	30 (44.12)	

Note:.

⁎ = More than one answer possible for every woman.

CH, FM, and HC present pain more often localized in the vaginal introitus, and the initial and the deep part of the vagina. CO group in addition reported pain in the external genitalia (more than 50 %). In the case of VU, the pain is more frequently localized in the vaginal introitus, the initial part of the vagina, the external genitalia, and the clitoris.

Regarding the duration of the pain expressed in number of months since the onset (Table 2), CO group reported having pain for significantly longer than CH and HC (*F* = 3.054; df = 4; *p* = 0.017). More often the pain is described as brief momentary transient by CH and HC, as continuous steady constant by FM and CO, and rhythmic periodic intermittent by VU. In most cases, clinical groups compared to controls report pain with intercourse requiring interruption or discontinuance, especially in the case of VU followed by CO. In addition, nearly 80 % of the VU group is found to have a clinical score at FSFI indicating the presence of sexual dysfunction, followed by 75.69 % of CO, 65.93 % of FM and 53.33 % of CH. In the current study, also the control group reports a notably percentage of FSFI clinical score at 44.12 %.

**Fig. 1:**
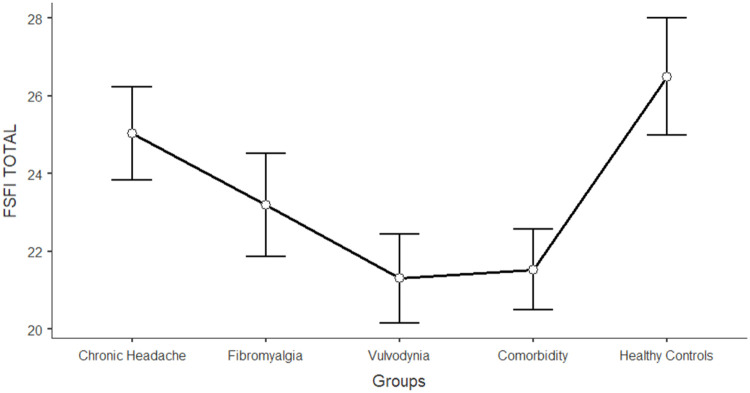
Differences between CH, FM, VU, CO, and HC groups on FSFI total: One-way ANCOVA.

Note: Lower scores in FSFI total indicates higher level of general sexual impairment

**Fig. 2:**
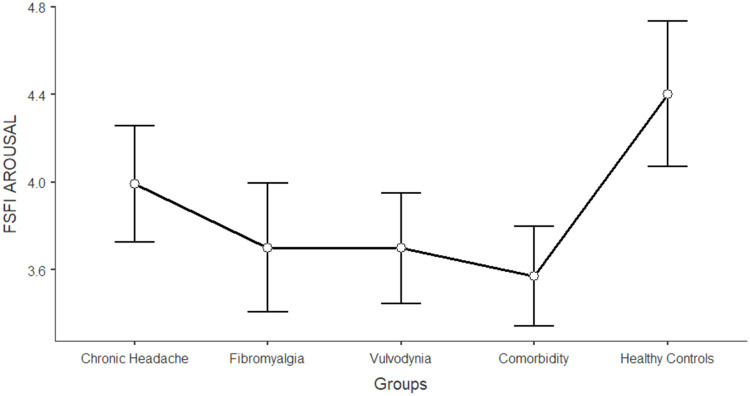
Differences between CH, FM, VU, CO, and HC groups on FSFI arousal: One-way ANCOVA.

Note: Lower scores in FSFI arousal indicates higher level of sexual impairment in this domain

**Fig. 3:**
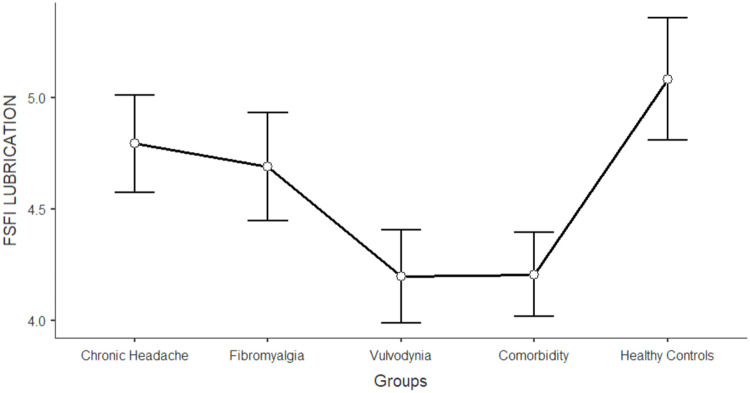
Differences between CH, FM, VU, CO, and HC groups on FSFI lubrication: One-way ANCOVA.

Note: Lower scores in FSFI lubrication indicates higher level of sexual impairment in this domain

**Fig. 4:**
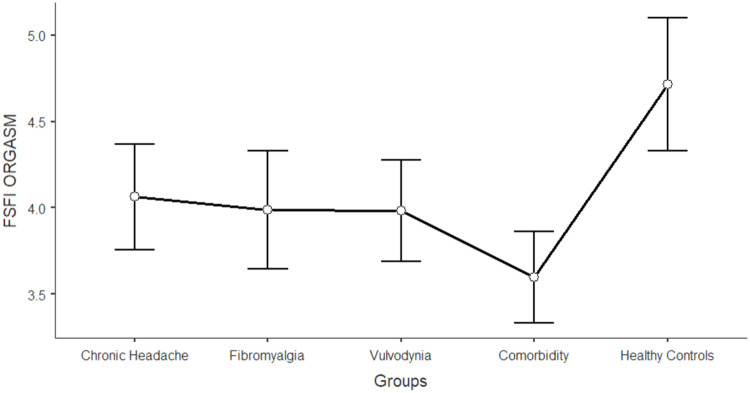
Differences between CH, FM, VU, CO, and HC groups on FSFI orgasm: One-way ANCOVA.

Note: Lower scores in FSFI orgasm indicates higher level of sexual impairment in this domain

**Fig. 5:**
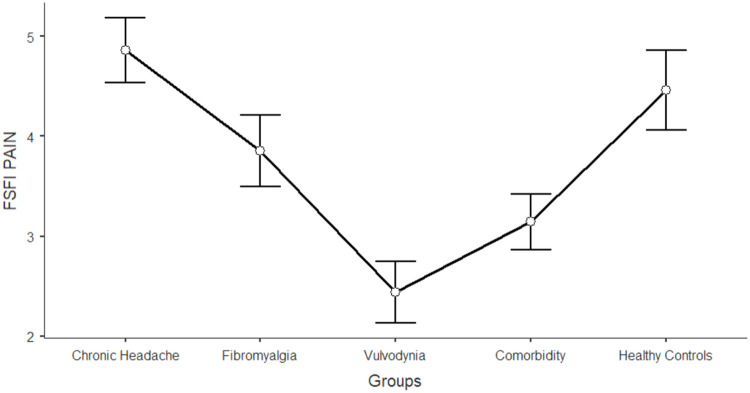
Differences between CH, FM, VU, CO, and HC groups on FSFI pain: One-way ANCOVA.

Note: Lower scores in FSFI pain indicates higher level of sexual impairment in this domain

**Fig. 6:**
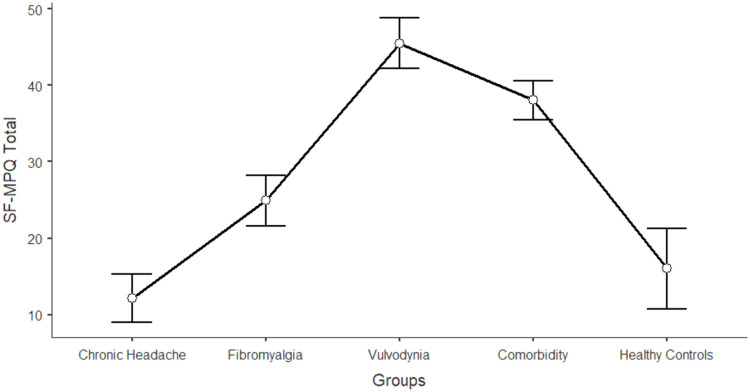
Differences between CH, FM, VU, CO, and HC groups on SF-MPQ genital pain: One-way ANCOVA.

Note: Higher scores in SF-MPQ Total indicates higher level of genito-pelvic pain

**Fig. 7:**
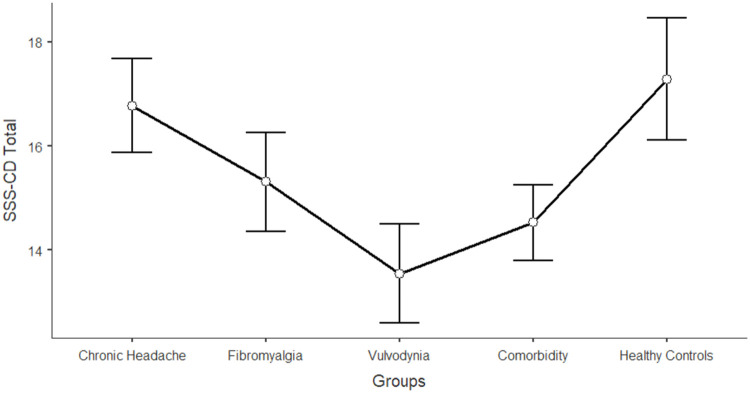
Differences between CH, FM, VU, CO, and HC groups on SSS-CD contentment: One-way ANCOVA.

Note: Higher scores in SSS-CD total indicates higher level of sexual satisfaction

The differences between women in the five groups in the domains of sexual functioning, genital pain, and sexual satisfaction were explored by running one-way ANCOVAs, with age as a covariate (see Table 3). Regarding sexual functioning, FM, VU, and CO generally reported significantly lower scores than HC and CH (*F* = 11.317; df = 4; *p* < 0.001). Sexual pain, as a domain of sexual functioning, explains the higher rate of variance among groups (22.3 %). In general, VU and CO reported significantly lower scores (indicating worse functioning) in sexual pain, arousal, lubrication, and orgasm. Compared to HC and CH, FM reported lower scores on sexual pain (indicating a higher presence of pain during sexual intercourse), but still higher than VU and CO scores. Figs. 1, 2, 3, 4, 5, 6, 7 depict the graphical representation of the ANCOVAs when Bonferroni's Post Hoc tests were significant. Regarding genital pain assessed with the adapted version of the SF-MPQ, which refers to the presence of pain in the genito-pelvic area associated or not with sexual activity, VU reported the highest scores indicating more pain, followed by CO, FM, and the other groups (*F* = 53.599; df = 4; *p* < 0.001). Notably, CH and HC did not report significant differences. This variable explains 24.8 % of the variance among groups. Individual sexual satisfaction was measured with the contentment domain of the SSS. Again, VU and CO reported significantly lower scores of satisfaction than the other groups (*F* = 11.858; df = 4; *p* < 0.001).

**Table 3:** Differences between CH, FM, VU, CO, and HC groups on sexual domains: One-way ANCOVAs.

	Chronic Headache (CH) (*n* = 105) *M*±DS	Fibromyalgia (FM) (*n* = 91) *M*±DS	Vulvodynia (VU) (*n* = 124) *M*±DS	Comorbidity (CO) (*n* = 144) *M*±DS	Healthy Controls (HC) (*n* = 68) *M*±DS	Post Hoc	F	*p*	Partial Eta^2^
**FSFI Total Score (Sexual Function)**	24.95±0.61	23.36±0.68	21.14±0.58	21.71±0.53	26.35±0.76	HC > FM, VU, CO CH >VU, CO	11.317	< 0.001	0.104
Desire	3.27±0.12	3.12±0.13	3.01±0.11	3.16±0.10	3.47±0.15	-*	7.237	< 0.001	0.006
Arousal	3.96±0.13	3.73±0.15	3.67±0.13	3.60±0.12	4.38±0.17	HC > VU, CO	7.767	< 0.001	0.071
Lubrication	4.79±0.11	4.70±0.12	4.19±0.11	4.21±0.10	5.08±0.14	CH, FM, HC > VU, CO	8.150	< 0.001	0.075
Orgasm	4.05±0.16	4.02±0.17	3.95±0.15	3.63±0.14	4.69±0.20	HC > VU, CO	4.631	< 0.001	0.039
Satisfaction	4.03±0.13	3.95±0.15	3.93±0.13	3.91±0.12	4.32±0.17	-*	3.437	0.002	0.027
Pain	4.84±0.16	3.89±0.18	2.40±0.16	3.19±0.14	4.42±0.20	FM, HC > VU, CO CH > FM > VU, CO	26.427	< 0.001	0.223
	Chronic Headache **(CH)** (*n* = 193) ***M*±DS**	Fibromyalgia (FM) (*n* = 185) ***M*±DS**	Vulvodynia (VU) (*n* = 193) ***M*±DS**	Comorbidity (CO) (*n* = 314) ***M*±DS**	Healthy Controls (HC) (*n* = 71) ***M*±DS**				
**SF-MPQ Total Score (Genital Pain)**	12.62±1.60	24.47±1.68	45.98±1.68	34.65±1.28	16.30±2.67	VU > CO> FM, CH, HC FM > CH	53.599	< 0.001	0.248
	Chronic Headache **(CH)** (*n* = 195) ***M*±DS**	Fibromyalgia (FM) (*n* = 186) ***M*±DS**	Vulvodynia (VU) (*n* = 193) ***M*±DS**	Comorbidity (CO) (*n* = 315) ***M*±DS**	Healthy Controls (HC) (*n* = 117) ***M*±DS**				
**SSS-CD (Contentment Domain)**	16.64±0.46	15.47±0.48	13.39±0.48	14.68±0.37	17.09±0.60	CH, HC > VU, CO FM > VU	11.858	< 0.001	0.061

Note:.

⁎ = General model is significant with post hoc not significant.

Following the second and last aim of the study, the sexual predictors of central sensitization, physical, and mental QoL were assessed separately for each group with age as a covariate. Results are reported in Table 4. Regarding CSI, all groups except controls showed genital pain as the main predictor of CSI (with higher scores of genital pain associated to higher scores of CSI). Moreover, CH and FM also showed a significant role of sexual satisfaction in predicting CSI scores (with higher scores of satisfaction associated to lower scores of CSI). For physical QoL, only the VU and CO groups reported genital pain as a significant sexual predictor (with higher scores of genital pain associated to lower scores of QoL). When it comes to mental QoL, sexual satisfaction resulted in a significant predictor in CH and CO (with higher scores of satisfaction associated to higher scores of QoL), while genital pain and sexual satisfaction were significant for VU (higher scores of satisfaction and lower scores of genital pain associated to higher lower scores of QoL). No predictors emerged as significant for FM and HC.

## Discussion

To our knowledge, this study represents the first attempt to compare the experience of sexuality among women with various conditions attributed to nociplastic CP. Previous studies have typically examined sexual functioning by focusing on one specific condition or by grouping different diagnoses within the same category (e.g., chronic musculoskeletal pain) (Birke et al., 2019; Burri et al., 2014; Granero-Molina et al., 2023; Piarulli et al., 2021; Ricoy-Cano et al., 2022; Santos-Iglesias et al., 2022).

Part of the first aim focused on the presence and characteristics of genito-pelvic pain in the four groups compared with healthy controls from the general population. The first significant finding is the high prevalence of genital and sexual pain experienced by women in the various groups. The presence of this symptom in the VU group is not surprising, as genito-pelvic pain represents the main symptom of this condition (Bornstein et al., 2022; Connor et al., 2020). Consistent with the literature (Ghizzani et al., 2014; Granero-Molina et al., 2023), high levels were also found in FM and CO (which also includes comorbidities of FM and VU conditions). The percentage of pain in the CH group is comparable to that found by Gordon et al. (2014) (39 % vs 44 %), while that presented by the control group is similar to that reported by an earlier Italian study on the general population (Nimbi et al., 2020), that still remains high and worthy of attention. Regarding sexuality, as well explained by Basson's circular sexual response model (Basson, 2000), when pain becomes intertwined with the sexual response, it can detrimentally impact not only the entire sexual experience, but also psychological, relational, and social aspects. Genito-pelvic pain could have a significant and far-reaching impact, and adopting a holistic biopsychosocial approach, capable of addressing all the potential effects of pain, may represent the most effective treatment option for these women (Dewitte et al., 2018; Doggweiler et al., 2017; Meana et al., 2017).

The details of pain location also seem to indicate specificity with VU and CO reporting more external pain (vaginal introitus, and external genitalia) and the other groups with more internally localized pain. This could affect the sexual experience in various ways, hypothetically making even practices that do not involve penetration but do involve external stimulation more difficult. In any case, the literature on female sexuality explores little about the prevalence of and satisfaction with non-penetrative practices in women (Hämmerli et al., 2020; Mautz et al., 2023; Rossi et al., 2022). This could be the result of a bias related to gender sexual stereotypes, in which research has primarily focused on penetration (penetration primacy) as an expression of female sexual function (Nimbi et al., 2019, 2021). In addition, in a speculative way, it is reasonable to suppose that a specific focus on the external parts of the genitalia might be supported by the tendency of some VU women to live in an anticipatory way the anxiety related to painful intercourse. This anticipation could be also promoted by the pain catastrophizing, a well-studied construct in VU (Mautz et al., 2023).

The duration and description of painful sensations explored by the SF-MPQ also show significant differences. The duration of symptoms is certainly an element that in the literature shows a negative effect not only on sexuality, but also on psychophysical well-being in general (Chisari et al., 2021; Nimbi et al., 2020). As suggested earlier, Basson's model (Basson, 2000) can provide a realistic explanation for the deterioration of the sexual experience with the general practitioner, which occurs immediately after the first painful episode and continues to strengthen after each attempt (Rossi et al., 2020, 2021).

Indeed, we can see how clinical groups compared to controls report pain with intercourse requiring interruption or discontinuance, especially in the case of VU followed by CO, and how most of the participating women (including a substantial number of the HC women) report clinical levels at FSFI, indicating the presence of sexual dysfunction. In the latter case, we specify that the high presence of dysfunction in the controls may be since the internet-disseminated study may have attracted more attention from women sensitive to the issue of sexuality due to the presence of personal difficulties.

To deepen the first objective, we wanted to explore if there is a significant difference in sexual functioning, genital pain, and sexual satisfaction domains among the assessed groups. Regarding sexual functioning, FM, VU, and CO generally reported significantly lower scores than HC and CH, indicating the presence of sexual problems. The domain that contributed most to this difference was the presence of pain during penetrative intercourse (22.3 % variance). We emphasize for greater understanding that the FSFI was compiled only by women who had penetrative sex in the last 4 weeks (Rosen et al., 2000); therefore, this information is to be considered partial and mainly related to that subgroup of women who have sexual activity that contemplates penetration. In any case, other areas of sexuality are found to be impaired such as arousal, lubrication and orgasm in VU and CO. This does not mean that the other groups have good sexual function; on the contrary. It might be more likely that in women with VU there is a greater take-up of the sexual health aspect, since the symptom for which they seek health care is mainly located in the genital area, whereas in the case of FM and CH, sexual health remains more often overlooked.

It is also important to keep this in mind when analysing data on genito-pelvic pain (so not only caused during or because of sexual activity) in which FM also reports a significantly high score along with VU and CO. And the same goes for sexual satisfaction (Connor et al., 2020), which is significantly lower in the case of VU and CO.

The last objective was to investigate the role of sexual variables (functioning, pain, and satisfaction) in predicting the levels of central sensitization, physical, and mental QoL separately for each group. The data show that genital pain is the main predictor of CSI scores. In addition to that, for CH and FM a significant role of sexual satisfaction was also shown. This is particularly interesting and innovative if we consider CSI primarily as an expression of psychological hypervigilance and burden than increased responsiveness of nociceptive neurons (Adams et al., 2023). In this sense, data from this study could suggest a possible role of sexual health in increasing or mitigating the effects of psychological and physical distress experienced by women with CP (Breton et al., 2008; Edwards et al., 2020). It might be interesting for future studies, to see if integrated treatments working directly on improving the experience of genito-pelvic pain and sexual satisfaction may also improve the psychological burden attributable to CP (Pukall et al., 2020). It could also be extremely suggested that women who present with a high score at the CSI when being clinically evaluated for CP should also investigate sexual health aspects in depth, to accommodate the woman with an increasingly holistic and inclusive approach to health (McGrath et al., 2021; Nimbi et al., 2021).

Regarding QoL, on the one hand, only the VU and CO groups reported genital pain as a significant sexual predictor of physical QoL. On the other hand, when it comes to mental QoL, sexual satisfaction resulted in a significant predictor in CH and CO, while genital pain and sexual satisfaction were significant for VU only. This finding also seems to be in line with the association between sexual health and QoL, especially from a psychological perspective (Breton et al., 2008; Edwards et al., 2020; Enzlin & Clippeleir, 2011; Flynn et al., 2016; Forbes et al., 2017). In the case of FM, where no sexual predictor is significantly associated with QoL, perhaps it is worth broadening the focus and investigating the role of other biopsychosocial aspects that might have a greater impact on QoL than sexuality. For example, Offenbaecher et al. (2021) showed that the major predictors of QoL in patients with FM in their study were depression, pain-related interference with everyday life, general activity, general health perception, punishing response from others, work status, vitality, and cognitive difficulties.

In clinical practice, it should be strongly suggested that any woman complaining of genital problems seek help immediately after the first onset of painful symptoms. It is recommended that the CP clinician may ask women with respect to the experience of pain. Women may find it difficult to report these kinds of symptoms, especially when the main diagnosis is FM or CH (McGrath et al., 2021; Nimbi et al., 2021). In addition, not only the time and location, but also the description of pain (e.g., brief, momentary, transient; continuous, steady, constant; and rhythmic, periodic, intermittent) may be a variable to consider maximizing the effectiveness of the diagnostic process and treatment (Vasileva et al., 2020). This may help to foster the access to care for these women, before the effects of pain cause clinically significant outcomes to their sexual health and QoL.

It might be useful to emphasize to the reader how much a sex-positive approach (Nimbi et al., 2021) may help these women in exploring a sexuality that also contemplates non-penetrative experiences (especially if this practice is painful) as an enriching and fulfilling component. Some clinical recommendation (Dewitte & Reisman, 2021; Mona et al., 2014) also suggest the involvement of use of sex toys and certain positions to adopt in sexual intercourse as an element that can foster a better experience of sexuality. Obviously, this step is possible where the willingness of the woman with CP to improve her sexual well-being is present.

To provide a more comprehensive understanding of the study, it is essential to discuss the inherent limitations of the research design:

(i) The administration of questionnaires was conducted online, utilizing snowball sampling for the survey's dissemination. While the cohort of women was reached through patients' associations, it cannot be assumed that all participants were diagnosed following CP criteria. The etiopathogenesis of CP was not verified through direct diagnostic examinations but solely self-reported by participants. Consequently, there may be concerns regarding the generalizability of the results. In essence, while self-report data has provided a foundational understanding of differences in sexual experiences among CP conditions, future research may require more rigorous experimental control to isolate specific diagnoses.
(ii) Opting for a web-based survey may have limited the participation of women less familiar and confident with technology.
(iii) The protocol solely comprised self-report questionnaires. Future studies should supplement these measures with direct physical assessments (such as the tampon test, pelvic floor examination, etc.), in-person interviews, focus groups, and the inclusion of partners to consider the relational aspects of sexuality in women with CP (Wammen Rathenborg et al., 2019).
(iv) Although an open question on medication was inserted in the protocol, the variety and discontinuity of the answer received did not allow us to classify this variable for the different types of medication in a way that was usable in the analysis (some women answered only “yes”, others reported many different classes of medication, 107 participants did not answer at all). This did not make it possible to determine the specific weight of pharmacological treatments on sexuality in the different groups examined, an element that needs further investigation in the future.

## Conclusion

This study shows that in all groups of women with CP, sexuality is a damaged area and there is a higher presence of genito-pelvic pain than in the healthy control group. Therefore, it is important to emphasize the need to work on two basic aspects: on the one hand, the possibility of investigating the presence of genito-pelvic pain and sexual difficulties in the diagnostic or usual care setting of the patient with CP (Flegge et al., 2023). On the other, to alleviate the symptomatology related to genito-pelvic pain and increase satisfaction rather than focusing only on sexual function (Nimbi et al., 2021). This would also allow a shift away from a purely performative style geared toward sexual function rather than well-being and quality of life.

In addition, some differences between the groups emerged and suggest to us that where help on sexuality and sexual pain on VU and CO seems primary and necessary, an analysis of the state of sexual health in FM and CH still remains highly recommended.

## Institutional review board statement

The study was conducted in accordance with the Declaration of Helsinki and approved by the Institutional Ethics Committee of the Department of Dynamic and Clinical Psychology and Health Studies, Sapienza University of Rome on November 25, 2022 [Protocol number 0001979 UOR: SI000092—Classified VII/15].

## Informed consent statement

Informed consent was obtained from all subjects involved in the study.

## Data availability statement

Data are available on request to the corresponding author.

## Declaration of generative AI and AI-assisted technologies in the writing process’

During the preparation of this work the author(s) used ChatGPT v.3.5 in order to proofread the English text. After using this tool/service, the author(s) reviewed and edited the content as needed and take full responsibility for the content of the publication.

## Declaration of competing interest

The authors declare that they have no known competing financial interests or personal relationships that could have appeared to influence the work reported in this paper.

## Appendix Supplementary materials

application 1 Image, application 1

## Footnotes

Supplementary material associated with this article can be found, in the online version, at doi:10.1016/j.ijchp.2024.100472.

## References

[bib0001] Adams G.R., Gandhi W., Harrison R., van Reekum C.M., Wood-Anderson D., Gilron I. (2023). Do “central sensitization” questionnaires reflect measures of nociceptive sensitization or psychological constructs? A systematic review and meta-analyses. Pain.

[bib0002] Basson R. (2000). The female sexual response: A different model. Journal of Sex & Marital Therapy.

[bib0003] Birke H., Ekholm O., Højsted J., Sjøgren P., Kurita G.P. (2019). Chronic pain, opioid therapy, sexual desire, and satisfaction in sexual life: A population-based survey. Pain Medicine.

[bib0004] Bornstein J., Goldstein A.T., Stockdale C.K., Bergeron S., Pukall C., Zolnoun D., International Society for the Study of Vulvovaginal Disease (ISSVD) (2016). 2015 ISSVD, ISSWSH, and IPPS consensus terminology and classification of persistent vulvar pain and vulvodynia. The Journal of Sexual Medicine.

[bib0005] Bornstein J., Palzur E., Swash M., Petros P. (2022). Vulvodynia: A neuroinflammatory pain syndrome originating in pelvic visceral nerve plexuses due to mechanical factors. Archives of Gynecology and Obstetrics.

[bib0006] Breton A., Miller C.M., Fisher K. (2008). Enhancing the sexual function of women living with chronic pain: A cognitive-behavioural treatment group. Pain Research & Management : The Journal of the Canadian Pain Society.

[bib0007] Burri A., Lachance G., Williams F.M.K. (2014). Prevalence and risk factors of sexual problems and sexual distress in a sample of women suffering from chronic widespread pain. The Journal of Sexual Medicine.

[bib0008] Caponnetto V., Deodato M., Robotti M., Koutsokera M., Pozzilli V., Galati C. (2021). Comorbidities of primary headache disorders: A literature review with meta-analysis. The Journal of Headache and Pain.

[bib0009] Casale R., Atzeni F., Bazzichi L., Beretta G., Costantini E., Sacerdote P. (2021). Pain in women: A perspective review on a relevant clinical issue that deserves prioritization. Pain and Therapy.

[bib0010] Chiarotto A., Viti C., Sulli A., Cutolo M., Testa M., Piscitelli D. (2018). Cross-cultural adaptation and validity of the Italian version of the central sensitization inventory. Musculoskeletal Science and Practice.

[bib0011] Chisari C., Monajemi M.B., Scott W., Moss-Morris R., McCracken L.M. (2021). Psychosocial factors associated with pain and sexual function in women with Vulvodynia: A systematic review. European Journal of Pain.

[bib0012] Cohen S.P., Vase L., Hooten W.M. (2021). Chronic pain: An update on burden, best practices, and new advances. The Lancet.

[bib0013] Collado-Mateo D., Olivares P.R., Adsuar J.C., Gusi N. (2020). Impact of fibromyalgia on sexual function in women. Journal of Back and Musculoskeletal Rehabilitation.

[bib0014] Connor J.J., Haviland M., Brady S.S., Robinson B.B.E., Harlow B.L. (2020). Psychosocial factors influence sexual satisfaction among women with vulvodynia. Journal of Sex & Marital Therapy.

[bib0015] Dagostin Ferraz S., Rodrigues Candido A.C., Rodrigues Uggioni M.L., Colonetti T., Santina Dagostin V., Rosa M.I. (2024). Assessment of anxiety, depression and somatization in women with vulvodynia: A systematic review and META-analysis. Journal of Affective Disorders.

[bib0016] Dahlhamer J., Lucas J., Zelaya C., Nahin R., Mackey S., DeBar L. (2018). Prevalence of Chronic Pain And High-Impact Chronic Pain Among Adults—United States, 2016. MMWR. Morbidity and Mortality Weekly Report.

[bib0017] Dewitte M., Borg C., Lowenstein L. (2018). A psychosocial approach to female genital pain. Nature Reviews. Urology.

[bib0018] Dewitte M., Reisman Y. (2021). Clinical use and implications of sexual devices and sexually explicit media. Nature Reviews Urology.

[bib0019] Doggweiler R., Whitmore K.E., Meijlink J.M., Drake M.J., Frawley H., Nordling J. (2017). A standard for terminology in chronic pelvic pain syndromes: A report from the chronic pelvic pain working group of the international continence society. Neurourology and Urodynamics.

[bib0020] Edwards S., Mandeville A., Petersen K., Cambitzi J., Williams A.C., de C. (2020). ‘ReConnect’: A model for working with persistent pain patients on improving sexual relationships. British Journal of Pain.

[bib0021] Enzlin P., Clippeleir D. (2011). The emerging field of «oncosexology»: Recognising the importance of addressing sexuality in oncology. Belgian Journal of Medical Oncology.

[bib0022] Filocamo M.T., Serati M., Li Marzi V., Costantini E., Milanesi M., Pietropaolo A. (2014). The Female Sexual Function Index (FSFI): Linguistic validation of the Italian version. The Journal of Sexual Medicine.

[bib0023] Finn E., Morrison T.G., McGuire B.E. (2018). Correlates of sexual functioning and relationship satisfaction among men and women experiencing chronic pain. Pain Medicine.

[bib0024] Fitzcharles M.A., Cohen S.P., Clauw D.J., Littlejohn G., Usui C., Häuser W. (2021). Nociplastic pain: Towards an understanding of prevalent pain conditions. The Lancet.

[bib0025] Flegge L.G., Barr A., Craner J.R. (2023). Sexual functioning among adults with chronic pain: Prevalence and association with pain-related outcomes. Pain Medicine.

[bib0026] Flynn K.E., Lin L., Bruner D.W., Cyranowski J.M., Hahn E.A., Jeffery D.D. (2016). Sexual satisfaction and the importance of sexual health to quality of life throughout the life course of U.S. adults. The Journal of Sexual Medicine.

[bib0027] Forbes M.K., Eaton N.R., Krueger R.F. (2017). Sexual quality of life and aging: A prospective study of a nationally representative sample. The Journal of Sex Research.

[bib0028] Galli F. (2023). Understanding nociplastic pain: Building a bridge between clinical psychology and medicine. Journal of Personalized Medicine.

[bib0029] Ghizzani A., Di Sabatino V., Suman A.L., Biasi G., Santarcangelo E.L., Carli G. (2014). Pain symptoms in fibromyalgia patients with and without provoked vulvodynia. Pain Research and Treatment.

[bib0030] Gordon A., Paneduro D., Pink L., Lawler V., Lay C. (2014). Evaluation of the frequency and the association of sexual pain and chronic headaches. Headache: The Journal of Head and Face Pain.

[bib0031] Granero-Molina J., Jiménez-Lasserrotte M., del M., Dobarrio-Sanz I., Correa-Casado M., Ramos-Rodríguez C. (2023). Sexuality in women with fibromyalgia syndrome: A metasynthesis of qualitative studies. Healthcare.

[bib0032] Hämmerli S., Kohl-Schwartz A., Imesch P., Rauchfuss M., Wölfler M.M., Häberlin F. (2020). Sexual satisfaction and frequency of orgasm in women with chronic pelvic pain due to endometriosis. The Journal of Sexual Medicine.

[bib0033] Harlow B.L., Kunitz C.G., Nguyen R.H.N., Rydell S.A., Turner R.M., MacLehose R.F. (2014). Prevalence of symptoms consistent with a diagnosis of vulvodynia: Population-based estimates from 2 geographic regions. American Journal of Obstetrics and Gynecology.

[bib0034] James S.L., Abate D., Abate K.H., Abay S.M., Abbafati C., Abbasi N. (2018). Global, regional, and national incidence, prevalence, and years lived with disability for 354 diseases and injuries for 195 countries and territories, 1990–2017: A systematic analysis for the Global Burden of Disease Study 2017. The Lancet.

[bib0035] Johnston, K.J.A., Signer, R., & Huckins, L.M. (2023). *Chronic overlapping pain conditions and nociplastic pain* (p. 2023.06.27.23291959). medRxiv. 10.1101/2023.06.27.23291959. 10.1016/j.xhgg.2024.100381 PMC11617767 39497418

[bib0036] Kosek E., Clauw D., Nijs J., Baron R., Gilron I., Harris R.E. (2021). Chronic nociplastic pain affecting the musculoskeletal system: Clinical criteria and grading system. Pain.

[bib0037] Lamvu G., Carrillo J., Ouyang C., Rapkin A. (2021). Chronic pelvic pain in women: A review. JAMA.

[bib0038] Marinoff S.C., Turner M.L. (1992). Vulvar vestibulitis syndrome. Dermatologic Clinics.

[bib0039] Marques A.P., Santo A.D.S.D.E., Berssaneti A.A., Matsutani L.A., Yuan S.L.K. (2017). Prevalence of fibromyalgia: Literature review update. Revista Brasileira de Reumatologia (English Edition).

[bib0040] Martinez-Lavin M. (2020). Fibromyalgia in women: Somatisation or stress-evoked, sex-dimorphic neuropathic pain?. Clinical and Experimental Rheumatology.

[bib0041] Mautz T.T., Mulroy M.E., Krapf J.M., Goldstein A.T., Pukall C.F. (2023). Pleasure despite pain: Associations between experiences of vulvar pleasure, vulvar pain, and sexual function in patients with chronic vulvar pain conditions. Sexual Medicine.

[bib0042] Mayer T.G., Neblett R., Cohen H., Howard K.J., Choi Y.H., Williams M.J. (2012). The development and psychometric validation of the central sensitization inventory. Pain Practice.

[bib0043] McGrath M., Low M.A., Power E., McCluskey A., Lever S. (2021). Addressing sexuality among people living with chronic disease and disability: A systematic mixed methods review of knowledge, attitudes, and practices of health care professionals. Archives of Physical Medicine and Rehabilitation.

[bib0044] Meana M., Fertel E., Maykut C. (2017). The wiley handbook of sex therapy.

[bib0045] Melzack R. (1987). The short-form McGill pain questionnaire. Pain.

[bib0046] Meston C., Trapnell P. (2005). Development and validation of a five-factor sexual satisfaction and distress scale for women: The Sexual Satisfaction Scale for Women (SSS-W). The Journal of Sexual Medicine.

[bib0047] Mona L.R., Syme M.L., Cameron R.P. (2014). Principles and practice of sex therapy, 5th ed.

[bib0048] Nappi R.E., Terreno E., Tassorelli C., Sances G., Allena M., Guaschino E. (2012). Sexual function and distress in women treated for primary headaches in a tertiary university center. The Journal of Sexual Medicine.

[bib0049] Nijs J., Lahousse A., Kapreli E., Bilika P., Saraçoğlu İ., Malfliet A. (2021). Nociplastic pain criteria or recognition of central sensitization? Pain phenotyping in the past, present and future. Journal of Clinical Medicine.

[bib0050] Nimbi F.M., Galizia R., Rossi R., Limoncin E., Ciocca G., Fontanesi L. (2021). The biopsychosocial model and the sex-positive approach: An integrative perspective for sexology and general health care. Sexuality Research & Social Policy: A Journal of the NSRC.

[bib0051] Nimbi F.M., Rossi R., Tripodi F., Wylie K., Simonelli C. (2019). A biopsychosocial model for the counseling of hormonal contraceptives: A review of the psychological, relational, sexual, and cultural elements involved in the choice of contraceptive method. Sexual Medicine Reviews.

[bib0052] Nimbi F.M., Rossi V., Tripodi F., Luria M., Flinchum M., Tambelli R. (2020). Genital pain and sexual functioning: Effects on sexual experience, psychological health, and quality of life. The Journal of Sexual Medicine.

[bib0053] Offenbaecher M., Kohls N., Ewert T., Sigl C., Hieblinger R., Toussaint L.L. (2021). Pain is not the major determinant of quality of life in fibromyalgia: Results from a retrospective “real world” data analysis of fibromyalgia patients. Rheumatology International.

[bib0054] Piarulli A., Conversano C., Ciacchini R., Miniati M., Marchi L., Bazzichi L. (2021). Catastrophisation, chronic pain and sexuality: A cross-sectional investigation in fibromyalgia and rheumatoid arthritis. Clinical and Experimental Rheumatology.

[bib0055] Pukall C.F., Bergeron S., Rosen N.O., Jackowich R. (2020). Persistent genitopelvic pain: Classification, comorbidities, chronicity, and interpersonal factors. Current Sexual Health Reports.

[bib0056] Raja S.N., Carr D.B., Cohen M., Finnerup N.B., Flor H., Gibson S. (2020). The revised international association for the study of pain definition of pain: Concepts, challenges, and compromises. Pain.

[bib0057] Ricoy-Cano A.J., Cortés-Pérez I., del Carmen Martín-Cano M., De La Fuente-Robles Y.M. (2022). Impact of fibromyalgia syndrome on female sexual function: A systematic review with meta-analysis. JCR: Journal of Clinical Rheumatology.

[bib0058] Rosen C.B., Heiman J., Leiblum S., Meston C., Shabsigh R., Ferguson D. (2000). The Female Sexual Function Index (FSFI): A multidimensional self-report instrument for the assessment of female sexual function. Journal of Sex & Marital Therapy.

[bib0059] Rossi V., Galizia R., Tripodi F., Simonelli C., Porpora M.G., Nimbi F.M. (2022). Endometriosis and sexual functioning: How much do cognitive and psycho-emotional factors matter?. International Journal of Environmental Research and Public Health.

[bib0060] Rossi V., Tripodi F., Simonelli C., Galizia R., Nimbi F.M. (2021). Endometriosis-associated pain: A review of quality of life, sexual health and couple relationship. Minerva Obstetrics and Gynecology.

[bib0061] Rossi V., Viozzi E., Tripodi F., Porpora M.G., Simonelli C., Nimbi F.M. (2020). Endometriosis, sexuality and satisfaction: A pilot study on women with and without infertility. Sexologies.

[bib0062] Ruschak I., Montesó-Curto P., Rosselló L., Aguilar Martín C., Sánchez-Montesó L., Toussaint L. (2023). Fibromyalgia syndrome pain in men and women: A scoping review. Healthcare.

[bib0063] Santos-Iglesias P., Crump L., Henry J.L., LaChapelle D.L., Byers E.S. (2022). The sexual lives of women living with fibromyalgia: A qualitative study. Sexuality and Disability.

[bib0064] Sarzi-Puttini P., Giorgi V., Marotto D., Atzeni F. (2020). Fibromyalgia: An update on clinical characteristics, aetiopathogenesis and treatment. Nature Reviews Rheumatology.

[bib0065] Solmaz V., Ceviz A., Aksoy D., Cevik B., Kurt S., Gencten Y. (2016). Sexual dysfunction in women with migraine and tension-type headaches. International Journal of Impotence Research.

[bib0066] Stovner L.J., Hagen K., Linde M., Steiner T.J. (2022). The global prevalence of headache: An update, with analysis of the influences of methodological factors on prevalence estimates. The Journal of Headache and Pain.

[bib0067] Vasileva P., Strashilov S., Yordanov A. (2020). Aetiology, diagnosis, and clinical management of vulvodynia. Menopause Review/Przegląd Menopauzalny.

[bib0068] Wammen Rathenborg F.L., Zdaniuk B., Brotto L.A. (2019). What do different measures of pain tell us? A comparison in sexually active women with provoked vestibulodynia. The Journal of Sexual Medicine.

[bib0069] Ware J.E., Gandek B. (1998). Overview of the SF-36 health survey and the International Quality of Life Assessment (IQOLA) project. Journal of Clinical Epidemiology.

[bib0070] Ware J.E., Kosinski M., Keller S.D. (1996). A 12-item short-form health survey: Construction of scales and preliminary tests of reliability and validity. Medical Care.

[bib0071] Wolfe F., Ablin J., Guymer E.K., Littlejohn G.O., Rasker J.J. (2020). The relation of physical comorbidity and multimorbidity to fibromyalgia, widespread pain, and fibromyalgia-related variables. The Journal of Rheumatology.

[bib0072] Wolfe F., Clauw D.J., Fitzcharles M.A., Goldenberg D.L., Häuser W., Katz R.L. (2016). 2016 Revisions to the 2010/2011 fibromyalgia diagnostic criteria. Seminars in Arthritis and Rheumatism.

