# Supplementary material for: Role of sexuality in women with chronic pain: Results from an Italian cross-sectional study on chronic headache, fibromyalgia, and vulvodynia
Source: Int J Clin Health Psychol. 2024 Jun 7;24(2):100472. doi: 10.1016/j.ijchp.2024.100472 (PMC11214997; doi:10.1016/j.ijchp.2024.100472)
Supplement: application 1 [file mmc1.docx]

**Table 4. Multiple regression analysis separated for CH, FM, VU, CO and HC groups on sexual domains as predictors of Central Sensitization, Physical and Mental Quality of Life**

|  | **Chronic Headache**  **(CH) (n = 105)** | | | |  | **Fibromyalgia (FM) (n = 91)** | | | |  | **Vulvodynia (VU) (n = 124)** | | | |  | Comorbidity  (CO) (n = 144) | | | |  | **Healthy Controls (HC) (n = 68)** | | | |  |
| --- | --- | --- | --- | --- | --- | --- | --- | --- | --- | --- | --- | --- | --- | --- | --- | --- | --- | --- | --- | --- | --- | --- | --- | --- | --- |
| **Predictors of Central Sensitization (CSI)** | ***B*** | ***SE*** | ***t*** | ***p*** | ***r^2^ = 0.1928*** | ***B*** | ***SE*** | ***t*** | ***p*** | ***r^2^ = 0.2808*** | ***B*** | ***SE*** | ***t*** | ***p*** | ***r2 = 0.2180*** | ***B*** | ***SE*** | ***t*** | ***p*** | ***r2 = 0.1269*** | ***B*** | ***SE*** | ***t*** | ***p*** | ***r2 = 0.1644*** |
| Age (covariate) | 0.0261 | 0.1005 | 0.2598 | 0.796 |  | 0.0416 | 0.1031 | 0.4034 | 0.688 |  | 0.0558 | 0.1253 | 0.4451 | 0.657 |  | 0.1233 | 0.1126 | 1.0944 | 0.276 |  | 0.1431 | 0.2178 | 0.6571 | 0.515 |  |
| Sexual Functioning (FSFI Total) | 0.0589 | 0.2250 | 0.2619 | 0.794 |  | 0.2464 | 0.2483 | 0.9923 | 0.324 |  | -0.1589 | 0.3126 | -0.5084 | 0.612 |  | -0.1527 | 0.3009 | -0.5074 | 0.613 |  | -0.2072 | 0.7350 | -0.2818 | 0.780 |  |
| Genital Pain (McGill Total) | 0.2330 | 0.0672 | 3.4681 | < .001 | *** | 0.2246 | 0.0487 | 4.6138 | < .001 | *** | 0.3729 | 0.0812 | 4.5935 | < .001 | *** | 0.1782 | 0.0569 | 3.1303 | 0.002 | ** | 0.2085 | 0.1722 | 1.2110 | 0.233 |  |
| Sexual Satisfaction (SSS - CD) | -0.4864 | 0.2033 | -2.3932 | 0.019 | * | -0.6045 | 0.2398 | -2.5211 | 0.014 | * | -0.3538 | 0.2967 | -1.1922 | 0.236 |  | -0.1745 | 0.2868 | -0.6085 | 0.544 |  | -0.3737 | 0.5219 | -0.7161 | 0.478 |  |
|  |  |  |  |  |  |  |  |  |  |  |  |  |  |  |  |  |  |  |  |  |  |  |  |  |  |
| **Predictors of Physical Quality of Life (SF-12)** | ***B*** | ***SE*** | ***t*** | ***p*** | ***r2 = 0.0420*** | ***B*** | ***SE*** | ***t*** | ***p*** | ***r2 = 0.0599*** | ***B*** | ***SE*** | ***t*** | ***p*** | ***r2 = 0.2898*** | ***B*** | ***SE*** | ***t*** | ***p*** | ***r2 = 0.0687*** | ***B*** | ***SE*** | ***t*** | ***p*** | ***r2 = 0.1895*** |
| Age (covariate) | -0.0902 | 0.1519 | -0.5941 | 0.554 |  | -0.1042 | 0.1226 | -0.8494 | 0.398 |  | -0.1482 | 0.1195 | -1.2401 | 0.217 |  | -0.0875 | 0.1132 | -0.7729 | 0.441 |  | 0.0535 | 0.1900 | 0.2818 | 0.780 |  |
| Sexual Functioning (FSFI Total) | -0.2704 | 0.3399 | -0.7956 | 0.428 |  | 0.0678 | 0.2955 | 0.2294 | 0.819 |  | -0.1900 | 0.2981 | -0.6372 | 0.525 |  | 0.2437 | 0.3026 | 0.8053 | 0.422 |  | 0.7902 | 0.6410 | 1.2327 | 0.225 |  |
| Genital Pain (McGill Total) | -0.1143 | 0.1015 | -1.1261 | 0.263 |  | -0.0160 | 0.0579 | -0.2759 | 0.783 |  | -0.4871 | 0.0774 | -6.2918 | < .001 | *** | -0.1345 | 0.0572 | -2.3500 | 0.020 | * | -0.0782 | 0.1502 | -0.5209 | 0.605 |  |
| Sexual Satisfaction (SSS - CD) | 0.5211 | 0.3071 | 1.6968 | 0.093 |  | 0.3485 | 0.2853 | 1.2215 | 0.225 |  | 0.4201 | 0.2830 | 1.4844 | 0.140 |  | -0.1562 | 0.2884 | -0.5417 | 0.589 |  | 0.1472 | 0.4552 | 0.3234 | 0.748 |  |
|  |  |  |  |  |  |  |  |  |  |  |  |  |  |  |  |  |  |  |  |  |  |  |  |  |  |
| **Predictors of Mental Quality of Life (SF-12)** | ***B*** | ***SE*** | ***t*** | ***p*** | ***r2 = 0.1172*** | ***B*** | ***SE*** | ***t*** | ***p*** | ***r2 = 0.0459*** | ***B*** | ***SE*** | ***t*** | ***p*** | ***r2 = 0.1960*** | ***B*** | ***SE*** | ***t*** | ***p*** | ***r2 = 0.1069*** | ***B*** | ***SE*** | ***t*** | ***p*** | ***r2 = 0.2370*** |
| Age (covariate) | 0.1808 | 0.1514 | 1.1938 | 0.235 |  | -0.1352 | 0.1272 | -1.0629 | 0.291 |  | 0.3227 | 0.1338 | 2.4116 | 0.017 | * | 0.0387 | 0.1164 | 0.3329 | 0.740 |  | 0.1881 | 0.2146 | 0.8766 | 0.386 |  |
| Sexual Functioning (FSFI Total) | 0.0804 | 0.3390 | 0.2372 | 0.813 |  | -0.1501 | 0.3064 | -0.4899 | 0.625 |  | -0.0194 | 0.3338 | -0.0582 | 0.954 |  | -0.2074 | 0.3110 | -0.6669 | 0.506 |  | 0.1349 | 0.7239 | 0.1863 | 0.853 |  |
| Genital Pain (McGill Total) | -0.1233 | 0.1012 | -1.2182 | 0.226 |  | 0.0077 | 0.0601 | 0.1285 | 0.898 |  | -0.2345 | 0.0867 | -2.7051 | 0.008 | ** | -0.0257 | 0.0588 | -0.4376 | 0.662 |  | -0.1060 | 0.1696 | -0.6248 | 0.536 |  |
| Sexual Satisfaction (SSS - CD) | 0.6522 | 0.3062 | 2.1300 | 0.036 | * | 0.4276 | 0.2959 | 1.4450 | 0.152 |  | 0.6587 | 0.3169 | 2.0785 | 0.040 | * | 0.9111 | 0.2964 | 3.0738 | 0.003 | ** | 0.8895 | 0.5140 | 1.7306 | 0.092 |  |
